# Two Randomized Trials of the Effect of Live Attenuated Influenza Vaccine on Pneumococcal Colonization

**DOI:** 10.1164/rccm.201811-2081LE

**Published:** 2019-05-01

**Authors:** Jamie Rylance, Wouter A. A. de Steenhuijsen Piters, Michael J. Mina, Debby Bogaert, Neil French

**Affiliations:** ^1^ Liverpool School of Tropical Medicine Liverpool, United Kingdom; ^2^ University of Edinburgh Edinburgh, United Kingdom; ^3^ University Medical Center Utrecht Utrecht, the Netherlands; ^4^ University of Liverpool Liverpool, United Kingdom and; ^5^ Harvard Medical School Boston, Massachusetts

*To the Editor*:

The human nasopharynx is frequently colonized by *Streptococcus pneumoniae* (the pneumococcus), serving as the reservoir for transmission, a state that necessarily precedes invasive pneumococcal infection. Influenza infection increases pneumococcal colonization density and dysregulates host immune responses, increasing the risk of secondary bacterial pneumonia and death (1–3).

Live attenuated influenza vaccine (LAIV) nasal spray has been used in the United States since 2003, and it has reduced severe influenza disease in the United Kingdom since its introduction in 2013 into the national pediatric immunization program. In mice, LAIV vaccination increases the density and duration of pneumococcal colonization (2) and rates of otitis media. In children, LAIV is associated with increased rates and density of bacterial colonization (4). Although LAIV is safe and not associated with increases in pneumococcal disease, these data suggest that it could increase pneumococcal transmission to susceptible individuals (5).

We therefore undertook two trials (EudraCT 2014-004634-26) using an established human challenge model to evaluate the effects of LAIV on the dynamics of pneumococcal colonization. Some of the results of these studies have been previously reported in the form of a preprint (https://doi.org/10.1101/343319). An extensive immunological investigation to accompany these clinical data has been published (6). Healthy nonsmoking volunteers, 18–50 years old, consented to participate in double-blinded, randomized, placebo-controlled trials reflecting alternative scenarios: *1*) immunization first (LAIV precedes nasopharyngeal inoculation with pneumococcus by 3 days) and *2*) colonization first (LAIV is administered 3 days after colonization with pneumococcus). The participants, who were uncolonized at baseline, randomly received either intervention (nasal LAIV paired with intramuscular placebo of normal saline; AstraZeneca) or control (nasal placebo of normal saline paired with intramuscular influenza vaccination [Fluarix Tetra; GlaxoSmithKline]) with concealment by blindfolding. All of the participants gave written informed consent, with approval from the North West NHS Research Ethics Committee (14/NW/1460).

All of the participants were inoculated with *S. pneumoniae* serotype 6B strain BHN418 (80,000 cfu per nostril) in 0.1 ml solution (7). (The *S. pneumoniae* BHN418 sequence [GI:557376079] is available from https://www.ncbi.nlm.nih.gov/nuccore/557376079). “Colonization positivity” was determined by serial nasal washes and defined by detection of serotype 6B by culture at a programmed time point from 2 to 29 days (7, 8). In parallel, PCR detection of pneumococcal *lytA* was performed. In the “immunization first” study, LAIV vaccination preceded pneumococcal inoculation by 3 days (primary endpoint: colonization rate). This order was reversed for the “colonization first” study (primary endpoint: area under the curve [AUC] of bacterial density between Days 2 and 14). Results are presented as modified intention to treat, excluding those who did not receive immunization or inoculation per protocol, or did not complete follow-up. Generalized linear models were used to compare colonization positivity, duration of colonization, and AUC bacterial density, with generalized estimating equations used for comparison at multiple time points. Full methodological and other details are available online in the form of a preprint (https://doi.org/10.1101/343319).

In the “immunization first” study (Figure 1), we enrolled 202 participants; 130 of these subjects were inoculated and 117 were analyzed (*n* = 55 LAIV, *n* = 62 control; overall mean age, 20 yr [range, 18–48 yr]; 58% female). Pneumococcal colonization rates were similar in LAIV participants and control subjects (25/55 [45.5%] vs. 24/62 [38.7%]; odds ratio [OR], 1.32; *P* = 0.46), although the LAIV-treated group had consistently yet nonsignificantly higher rates at each time point. PCR detection rates were significantly higher in the LAIV group than in the control group at Day 2 (33/55 [60.0%] vs. 25/62 [40.3%]; OR, 2.22; *P* = 0.03). The median duration of colonization was not different between the groups by conventional microbiology (22 d [interquartile range (IQR), 22–29] and 22 d [IQR, 14–29] in the LAIV and control groups, respectively; *P* = 0.09) or PCR (median, 22 d [IQR, 7–29] LAIV vs. 14 d [IQR, 7–22] control; *P* = 0.45). Mean colonization densities were consistently increased in the LAIV group, with statistical significance at Day 9 representing a 10-fold (1 log_10_) increase in colonization density in the LAIV group (2.82 ± 1.78 vs. 1.81 ± 1.39 log_10_ titers, *P* = 0.03; Figure 1). PCR results showed the same pattern, with significantly higher densities in the LAIV group at Day 2 (*P* = 0.03).

**Figure 1.**
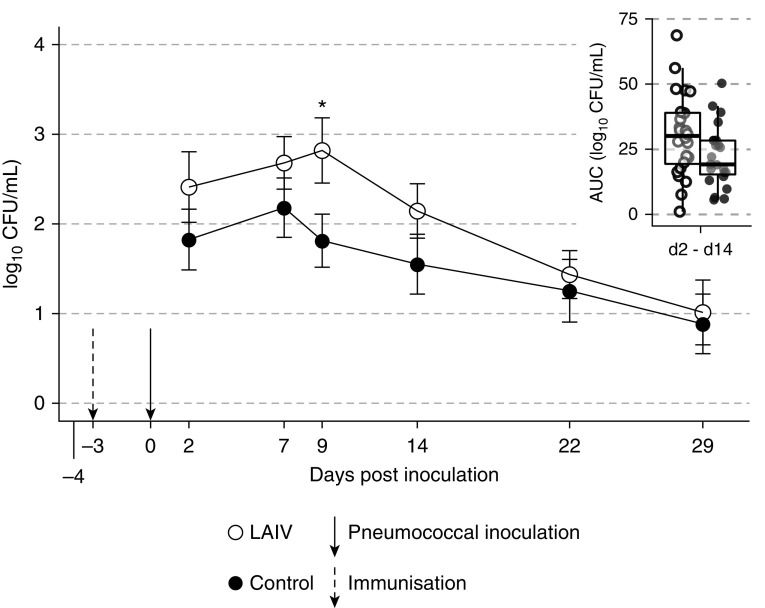
Immunization first: live attenuated influenza vaccine (LAIV) precedes nasopharyngeal inoculation with pneumococcus—effect on colonization dynamics of LAIV vaccination given at Day −3. Density dynamics after pneumococcal inoculation (on Day 0) are calculated from classical microbiology [log_10_(cfu/ml + 1)]. Mean density of *Streptococcus pneumoniae* for each nasal wash time point among participants in whom serotype 6B was detectable at any point. Bars represent SE. Inset: area under the curve (AUC) of density–time from Day 2 to Day 14 (box plot of median with interquartile range, with whiskers at 1.5× the interquartile range). *Statistically significant difference (*P* < 0.05).

Four participants with laboratory-confirmed other viral infections (three influenza B in the control arm, one rhinovirus in the LAIV arm) had among the highest bacterial densities of their cohorts. Among pneumococcal-colonized individuals, the AUC of colonization density was higher in the LAIV group than in the control group, with borderline statistical significance at Days 2–14 (*P* = 0.05), and reached statistical significance after exclusion of participants who had nasal-swab PCR evidence of concurrent wild-type viral illness (three influenza B in the control arm, one rhinovirus in the LAIV arm; data not shown; *P* = 0.03) after presenting with symptoms of illness.

In the “colonization first” study (Figure 2), 316 participants consented, 206 were screened, and 163 participants were included in the modified intention-to-treat analysis (*n* = 73 LAIV, *n* = 90 control; overall mean age, 20 yr [range, 18–46 yr]; 55% female). Data from 17 participants (10%) were excluded owing to non-study-serotype *S. pneumoniae* colonization. AUC colonization densities for each time period were consistently lower in the LAIV group, although the difference was not statistically significant (*P* = 0.11 for Days 2–14 primary endpoint; Figure 2). By PCR, a significantly lower AUC was evident in the LAIV group compared with the control group on Days 2–27 (*P* = 0.03).

**Figure 2.**
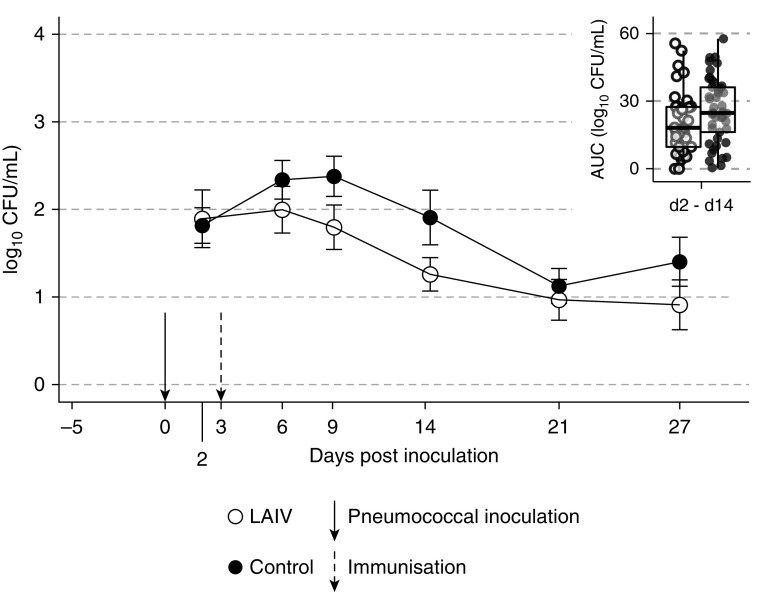
Colonization first: live attenuated influenza vaccine (LAIV) is administered to subjects already inoculated with pneumococcus—effect on colonization dynamics. Experimental inoculation to pneumococcus was performed on Day −3. Density dynamics after LAIV vaccination or control (on Day 0) are calculated from classical microbiology [log_10_(cfu/ml + 1)]. Mean density of *Streptococcus pneumoniae* for each nasal wash time point among participants in whom serotype 6B was detectable at any point. Bars represent SE. Inset: area under the curve (AUC) of density–time from Day 2 to Day 14 (the primary endpoint, box plot of median with interquartile range, with whiskers at 1.5× the interquartile range).

Rates of colonization did not differ between the LAIV and control groups by conventional microbiology (36/73 [49.3%] vs. 45/90 [50.0%] respectively; OR, 0.97; *P* = 0.93). The median colonization duration did not differ between the two groups (21 vs. 27 d, *P* = 0.17) by conventional microbiology, although it was lower in the LAIV group by PCR (14 vs. 27 d, *P* = 0.001).

There were no serious adverse events related to the intervention in either study.

In the largest trial to date involving a controlled human coinfection model, we have studied for the first time the impact of coinfection of a live viral vaccine and a bacterial pathogen. Immunological parameters have been reported separately (6).

Antecedent LAIV administration caused modest but significant transient effects on pneumococcal colonization, in keeping with a pediatric randomized controlled trial that showed an increased pneumococcal density after LAIV (2). In our study, the inverse scenario (LAIV after pneumococcal colonization) was associated with reduced colonization density and colonization rates at Day 27, decreased AUC, and earlier bacterial clearance.

Our model, consistent with murine coinfection disease models, reinforces the notion that the precedence of pathogen exposure might determine disease outcome: pneumococcal infection after influenza might exacerbate disease, whereas pneumococcus infection preceding influenza might reduce mortality (9). We used complementary methods for bacterial detection: although PCR is more sensitive and could detect DNA in the absence of viable pathogen, the persistence beyond 2 days suggests lower-density colonization, which is unmeasurable by culture.

These studies were limited by size and the evaluation of a single pneumococcal serotype in healthy adults likely to have neutralizing influenza antibodies. Any effect of LAIV in children may therefore be more pronounced owing to lower antibody titers, increased viral shedding, and higher natural rates of pneumococcal colonization acquisition. Future vaccine studies should evaluate the effect on pathogens not directly targeted by the vaccine, including their onward transmission.

## Supplementary Material

Supplements

Author disclosures
