# Supplementary material for: Two Randomized Trials of the Effect of Live Attenuated Influenza Vaccine on Pneumococcal Colonization
Source: Am J Respir Crit Care Med. 2019 May 1;199(9):1160–3. doi: 10.1164/rccm.201811-2081LE (PMC6515882; doi:10.1164/rccm.201811-2081LE)
Supplement: Supplements [file rccm.201811-2081LE.html]

Two Randomized Trials of the Effect of Live Attenuated Influenza Vaccine on Pneumococcal Colonization | American Journal of Respiratory and Critical Care Medicine

- disclosures.pdf (139 KB)
